# Influences of assisted breathing and mechanical ventilator settings on tidal volume and alveolar pressures in acute respiratory distress syndrome: a bench study

**DOI:** 10.5935/0103-507X.20210084

**Published:** 2021

**Authors:** Renata Santos Vasconcelos, Raquel Pinto Sales, Juliana Arcanjo Lino, Luíza Gabriela de Carvalho Gomes, Nancy Delma Silva Vega Canjura Sousa, Liégina Silveira Marinho, Bruno do Valle Pinheiro, Marcelo Alcantara Holanda

**Affiliations:** 1 Universidade Federal do Ceará - Fortaleza (CE), Brazil.; 2 Pulmonary and Critical Care Division, Hospital Universitário, Universidade Federal de Juiz de Fora - Juiz de Fora (MG), Brazil.

**Keywords:** Respiration, artificial, Respiratory distress syndrome, Intermittent positive-pressure breathing, Ventilator-induced lung injury

## Abstract

**Objective:**

To evaluate the influences of respiratory muscle efforts and respiratory rate setting in the ventilator on tidal volume and alveolar distending pressures at end inspiration and expiration in volume-controlled ventilation and pressure-controlled ventilation modes in acute respiratory distress syndrome.

**Methods:**

An active test lung (ASL 5000™) connected to five intensive care unit ventilators was used in a model of acute respiratory distress syndrome. Respiratory muscle efforts (muscle pressure) were configured in three different ways: no effort (muscle pressure: 0cmH_2_O); inspiratory efforts only (muscle pressure:-5cmH_2_O, neural inspiratory time of 0.6s); and both inspiratory and expiratory muscle efforts (muscle pressure:-5/+5cmH_2_O). Volume-controlled and pressure-controlled ventilation modes were set to deliver a target tidal volume of 420mL and positive end-expiratory pressure of 10cmH_2_O. The tidal volume delivered to the lungs, alveolar pressures at the end of inspiration, and alveolar pressures at end expiration were evaluated.

**Results:**

When triggered by the simulated patient, the median tidal volume was 27mL lower than the set tidal volume (range-63 to +79mL), and there was variation in alveolar pressures with a median of 25.4cmH_2_O (range 20.5 to 30cmH_2_O). In the simulated scenarios with both spontaneous inspiratory and expiratory muscle efforts and with a mandatory respiratory rate lower than the simulated patient's efforts, the median tidal volume was higher than controlled breathing.

**Conclusion:**

Adjusting respiratory muscle effort and pulmonary ventilator respiratory rate to a value above the patient’s respiratory rate in assisted/controlled modes generated large variations in tidal volume and pulmonary pressures, while the controlled mode showed no variations in these outcomes.

## INTRODUCTION

Ventilator-induced lung injury (VILI) is an iatrogenic cause of pulmonary damage related to excessive mechanical stress and/or strain imposed on the lung tissue during mechanical ventilation (MV).^(^1^)^ It is of particular concern for patients with acute respiratory distress syndrome (ARDS), as they present with severe lung edema and inflammation. Furthermore, mechanical alterations are heterogeneously distributed inside the lung parenchyma in ARDS, thereby predisposing the alveoli and small airways to excessive distension or pressures during tidal breathing.^(^2^)^ Setting the tidal volume (VT) to 4 to 6mL/kg of predicted or ideal body weight and limiting the distending pressures - both plateau (< 28 - 30cmH_2_O) and, particularly, the driving pressure (< 15cmH_*2*_O) - during MV were associated with improved survival in ARDS.^(^3^,^4^)^ In fact, so-called protective ventilatory strategies may prevent or attenuate VILI by reducing both the stress and the strain on the lungs caused by MV.^(^5^,^6^)^ They are now the standard of care for the initial controlled MV of patients with ARDS.^(^1^)^ However, mortality rates remain high, ranging from 34% to 60%.^(^2^,^7^-^9^)^

Currently, there are no guidelines on ventilating ARDS patients with preserved respiratory drive and spontaneous respiratory efforts, i.e., assisted MV. Maintaining spontaneous breathing during MV may have beneficial effects, such as preventing diaphragmatic atrophy and dysfunction, avoiding respiratory monotony regarding VT variation, and recruiting juxta diaphragmatic alveoli, which usually collapse in severe ARDS.^(^10^)^ All these factors may contribute to the early liberation of the patient from the ventilator.^(^11^-^13^)^ On the other hand, assisted breathing during MV may result in higher VT and transpulmonary pressures, especially in areas close to collapsed alveoli, and may result in tidal recruitment and pendelluft ventilation, thus amplifying heterogeneous distensions of the lung parenchyma.^(^14^)^ Even in patients with good patient-ventilator synchrony, the target VT and the desired airway pressure limits may be frequently exceeded,^(^15^)^ thus compromising the effectiveness of protective ventilatory strategies. Furthermore, patient-ventilator asynchronies such as double triggering, also referred to as breath-stacking or ineffective efforts, may result in huge VT and transpulmonary pressures, which increase the risk of VILI.^(^14^,^16^-^19^)^ Researchers have found an association between patient-ventilator asynchronies and mortality in mechanically ventilated patients.^(^20^)^ On the other hand, controlled MV has been associated with VILI prevention or attenuation in experimental studies and, more importantly, with improved outcomes, including survival, in patients with moderate or severe ARDS.^(^19^)^ Three randomized controlled trials demonstrated the positive impact of early neuromuscular blockade in ARDS on functional parameters and mortality.^(^12^,^21^,^22^)^

Little attention has been given to the influences of inspiratory and expiratory muscle efforts, ventilatory modes - either volume-controlled ventilation (VCV) or pressure-controlled ventilation (PCV) - or the number of mandatory respiratory cycles (set respiratory rate - RR), all of which are combined, on VT and distending alveolar pressures during assisted MV. The main differences between VCV and PCV during assisted MV are the amount and type of flow delivered to the lungs, which may be higher with greater patient effort and exponential deceleration in the latter. In both modes, the set RR may cause patient-ventilator asynchronies when it is higher than the spontaneous RR of the patient. Therefore, we hypothesized that: first, assisted breaths invariably result in a VT higher than that in controlled breathing cycles and higher alveolar pressures, even in synchronic breathings; second, triggering and cycling asynchronies caused by setting the RR higher than the patient’s spontaneous RR, regardless of the ventilatory mode (VCV or PCV) or the intensive care unit (ICU) ventilator type, causes huge variations in VT and alveolar distending pressures; third, early active expiratory effort during inspiration may limit VT augmentation and the correspondent increase in alveolar distending pressures.

The main objectives were to test the above hypothesis by evaluating the influences of respiratory muscle efforts - both inspiratory and expiratory - and of the RR setting in the ventilator - above or below the patient’s RR - on VT and alveolar distending pressures at end inspiration and expiration in both VCV and PCV in a mechanical simulated model of ARDS.

## METHODS

This bench study was conducted at the Respiration Laboratory of the Department of Internal Medicine of the Medical School of the *Universidade Federal do Ceará*, Brazil.

### Simulated model

An ASL 5000™ mechanical simulator (IngMar Medical, Pittsburgh, EUA) was used. The respiratory model was configured to reproduce, as realistically as possible, the mechanical characteristics of an adult patient with moderate to severe ARDS with spontaneous breathing efforts.^(^23^-^25^)^ The following parameter settings were used: static compliance 25mL/cmH_2_O, and inspiratory airway resistance 10cmH_2_O/L/sec.^(^26^)^ Respiratory muscle efforts (muscle pressure - Pmus) were configured in three different ways: no effort (Pmus: 0cmH_2_O); inspiratory efforts only (Pmus:-5cmH_2_O, neural inspiratory time of 0.6s); and both inspiratory and expiratory muscle efforts (Pmus:-5/+5cmH_2_O, with neural inspiratory and expiratory times of 0.6s each).^(^15^)^ The simulated patient RR was set at 20 bpm.

### Intensive care unit ventilators

Five ICU ventilators were used: Esprit V-1000 (Respironics™, Murrysville, EUA), DX 3012 (Dixtal™, Buenos Aires, Argentina), Servo I (Maquet™; Solna, Sweden), Puritan-Bennet 840 (Covidien Mansfield, MA, USA), and Savina 300 (Drager™, Lübeck, Germany). All ventilators used dual limbs (inspiratory and expiratory circuits) connected to a Y-adapter and an orotracheal tube (I.D 8.0mm) with no humidification system.^(^26^,^27^)^

### Experimental protocol

The ICU ventilators were tested and calibrated according to their manufacturer’s recommendations. Volume-controlled ventilation and PCV were used. Volume-controlled ventilation was set to deliver a target VT of 420mL (6mL/kg for an IBW of 70kg) and an inspiratory time of 0.8s with a constant flow (square wave format) of 31L/min. Pressure-controlled ventilation was set to deliver a VT of 420mL, as in the VCV, by carefully titrating airway pressure above the positive end-expiratory pressure (PEEP) with the same inspiratory time of 0.8s. In both modes a PEEP of 10cmH_2_O and a pressure triggering sensitivity threshold of 2cmH_2_O below PEEP were set.^(^15^,^25^)^ In the Savina 300™ ventilator, the tests were also run with the AutoFlow® (AF) system in VCV mode (VCV-AF). In short, this system calculates the respiratory compliance in each breath and automatically delivers an initial inspiratory flow that equals the ratio of the target VT to the respiratory compliance.^(^28^)^ Another characteristic of this mode is that it allows spontaneous breathing during the breathing cycle, as the inspiratory and expiratory valves are kept open during the two phases of the breathing cycle.^(^29^)^

### Measurements and outcomes

Each simulated scenario was recorded after stabilization of the respiratory pattern, which usually occurred rapidly, in less than 3 to 5 minutes, as was expected for a mechanical simulation. Thereafter, five consecutive minutes of the simulation were continuously recorded. Then, 20 representative breaths were selected for off-line analysis using ASL 5000™ software (LabVIEW; National Instruments; Austin, TX, USA). In total, 1.100 breaths were analyzed (3 scenarios, 2 modes, 2 settings of the mandatory RR, 5 ventilators, AF, 20 breaths per each) for the following variables: 1) VT delivered to the lungs, 2) alveolar pressures at the end of inspiration, 3) alveolar pressures at end expiration (effective or total PEEP) and 4) the difference between the alveolar pressure and the Pmus (which was considered a surrogate for the transpulmonary pressure as there is no pleural pressure in the mechanical model).

**Figure 1:**
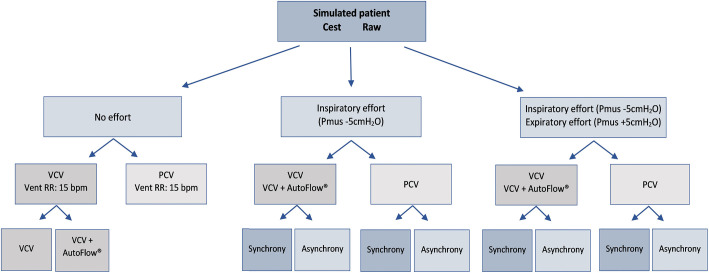
Simulated scenarios according to the ventilatory modes and settings of the mandatory respiratory rate and the presence and types of spontaneous respiratory efforts.

Figure 1 shows the simulated scenarios. A total of 10 scenarios were studied for each ventilator. For the Savina 300™ ventilator, one additional scenario was tested in the AF.

Cest - static compliance; Raw - airway resistance; Pmus - muscle pressure; VCV - volume-controlled ventilation; RR - respiratory rate; PCV - pressure-controlled ventilation.

**Figure 2:**
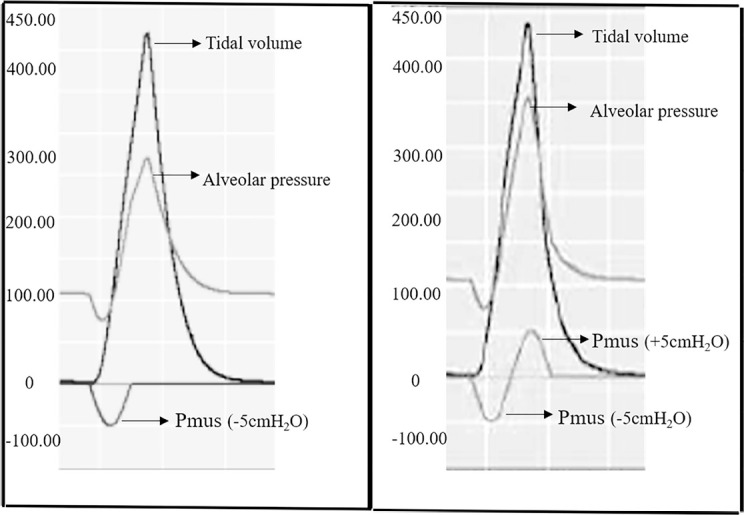
Two representative breathing cycles showing the tidal volume, alveolar pressure, and muscle pressure in the same plot.

Figure 2 depicts representative curves that show how the respiratory variables were measured.

The variables are described as medians and minimum and maximum values. Given the stability of the mechanical model and its almost negligible variability, we chose to make nominal comparisons between the obtained values without conducting comparative statistical tests.^(^24^,^26^)^ Differences that were considered potentially clinically relevant were highlighted and discussed. We predefined VT values > 560mL (8mL/kg) and end inspiration alveolar pressure (Palv) > 28cmH_2_O as clinically relevant.

## RESULTS

**Figure 3:**
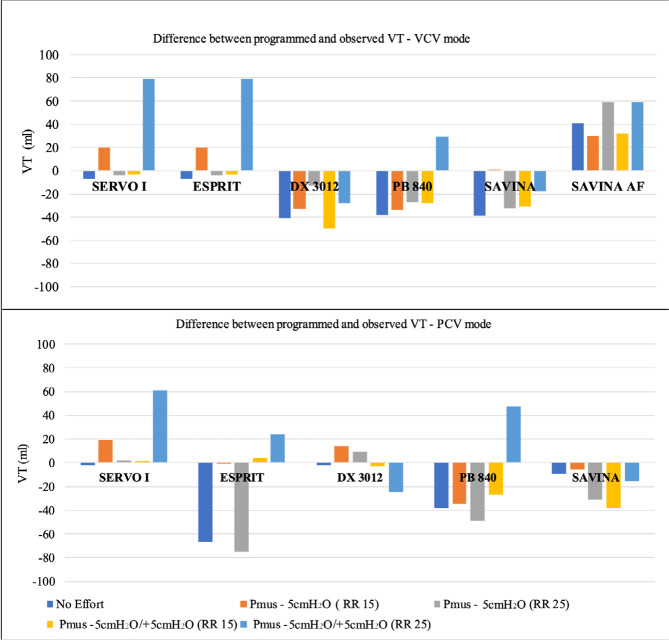
Difference between programmed and observed tidal volume in volume-controlled ventilation and pressure-controlled ventilation mode for the five ventilators in all simulated scenarios. The tidal volume programmed was 420mL.

Tables 1 to 4 show the results of the VT, Palv, total PEEP, and transpulmonary pressure end of inspiration for the five ventilators in all simulated scenarios, and figure 3 shows the difference between programmed (420mL) and observed VT in VCV and PCV mode. As expected, the VT remained constant with no variation during controlled MV (no effort, Pmus = 0). In general, during assisted MV, the VT, and alveolar pressures increased in both the VCV and PCV modes in all scenarios.

The Y axis depicts the absolute values used for the tidal volume (mL), and the pressure values (Palv and Pmus) are multiplied by 10, e.g., a value of 100.0 corresponds to 10cmH_2_O Pmus - muscle pressure.

**Table 1:** Tidal volume values in the volume-controlled ventilation and pressure-controlled ventilation modes for all the ventilators and simulated scenarios

VCV mode Ventilators ICU	VT (mL)						PCV mode	VT (mL)				
	Patient effort	No effort*	Pmus-5cmH_2_O		Pmus-5cmH_2_O/+5cmH_2_O			No effort*	Pmus-5cmH_2_O		Pmus-5cmH_2_O/+5cmH_2_O	
	Ventilator RR	zero	15	25	15	25		zero	15	25	15	25
SERVO I	Maximum	413 (-7)	440 (+20)	443 (+23)	417 (-3)	526 (+106)	SERVO I	418 (-2)	439 (+19)	483 (-63)	421 (+1)	546 (+126)
	Median	413 (-7)	440 (+20)	416 (-4)	417 (-3)	499 (+79)		418 (-2)	439 (+19)	422 (+2)	421 (+1)	481 (+61)
	Minimum	413 (-7)	440 (+20)	409 (-11)	417 (-3)	440 (+20)		418 (-2)	439 (+19)	413 (-7)	421 (+1)	476 (+56)
ESPRIT	Maximum	353 (-67)	358 (-62)	392 (-28)	362 (-58)	461 (+41)	ESPRIT	353 (-67)	419 (-1)	422 (+2)	424 (+4)	508 (+88)
	Median	353 (-67)	358 (-62)	363 (-57)	362 (-58)	413 (-7)		353 (-67)	419 (-1)	345 (-75)	424 (+4)	444 (+24)
	Minimum	353 (-67)	358 (-62)	356 (-64)	362 (-58)	367 (-53)		353 (-67)	419 (-1)	341 (79)	424 (+4)	430 (+10)
DX 3012	Maximum	379 (-41)	387 (-33)	422 (+2)	370 (-50)	406 (-14)	DX 3012	418 (-2)	434 (+14)	476 (+56)	417 (-3)	490 (+70)
	Median	379 (-41)	387 (-33)	407 (-13)	370 (-50)	392 (-28)		418 (-2)	434 (+14)	429 (+9)	417 (-3)	445 (-25)
	Minimum	379 (-41)	387 (-33)	394 (-26)	370 (-50)	370 (-50)		418 (-2)	434 (+14)	418 (-2)	417 (-3)	438 (+18)
PB 840	Maximum	382 (-38)	386 (-34)	416 (-4)	392 (-28)	474 (+54)	PB 840	382 (-38)	385 (-35)	424 (+4)	393 (-27)	536 (+116)
	Median	382 (-38)	386 (-34)	393 (-27)	392 (-28)	449 (+29)		382 (-38)	385 (-35)	371 (-49)	393 (-27)	467 (+47)
	Minimum	382 (-38)	386 (-34)	378 (-42)	392 (-28)	398 (-22)		382 (-38)	385 (-35)	363 (-57)	393 (-27)	448 (+28)
SAVINA	Maximum	381 (-39)	421 (+1)	421 (+1)	389 (-31)	463 (+43)	SAVINA	411 (-9)	414 (-6)	428 (+8)	382 (-38)	459 (+39)
	Median	381 (-39)	421 (+1)	388 (-32)	389 (-31)	402 (-18)		411 (-9)	414 (-6)	389 (-31)	382 (-38)	404 (-16)
	Minimum	381 (-39)	421 (+1)	367 (-53)	389 (-31)	400 (-20)		411 (-9)	414 (-6)	372 (-48)	382 (-38)	398 (-22)
SAVINA AF	Maximum	461 (+41)	450 (+30)	508 (+88)	452 (+32)	556 (+136)		-	-	-	-	-
	Median	461 (+41)	450 (+30)	479 (+59)	452 (+32)	479 (+59)						
	Minimum	461 (+41)	450 (+30)	430 (+10)	452 (+32)	468 (+48)						

VT - tidal volume; VCV - volume-controlled ventilation; ICU - intensive care unit; RR - respiratory rate; Pmus - muscle pressure; PCV - pressure-controlled ventilation.

* Absence of muscle effort: Pmus = zero. Inspiratory effort: Pmus =-5cmH_2_O, Expiratory effort: Pmus = +5cmH_2_O. The spontaneous respiratory rate of the patient was set at 20bpm, and the target tidal volume was set at 420mL. In parentheses is the difference between the programmed tidal volume and the observed tidal volume.

**Table 2:** Alveolar pressure at end-inspiration in the volume-controlled ventilation and pressure-controlled ventilation modes for all the ventilators and simulated scenarios

VCV mode Ventilators ICU	Palv						PCV mode	Palv				
	Patient effort	No effort*	Pmus-5cmH_2_O		Pmus-5cmH_2_O/+5cmH_2_O			No effort*	Pmus-5cmH_2_O		Pmus-5cmH_2_O/+5cmH_2_O	
	Ventilator RR	zero	15	25	15	25		zero	15	25	15	25
SERVO I	Maximum	26.2	27.3	27.4	31.3	32.7	SERVO I	26.4	27.3	27.2	31.3	31
	Median	26.2	27.3	26.4	31.3	25.5		26.4	27.3	26.4	31.3	26.3
	Minimum	26.2	27.3	22	31.3	22.9		26.4	27.3	24.8	31.3	24.2
ESPRIT	Maximum	24.2	24.7	25.9	29.7	30	ESPRIT	24.1	26.7	25.9	32.1	31.6
	Median	24.2	24.7	24.9	29.7	24.6	24.1	26.7	24.3	32.1	25.9	
	Minimum	24.2	24.7	21.3	29.7	23	24.1	26.7	22.5	32.1	24.3	
DX 3012	Maximum	25.2	25.5	22.2	30	30.6	DX 3012	26.8	27.4	27.4	31.7	31.8
	Median	25.2	25.5	26.1	30	24.5		26.8	27.4	26.7	31.7	26.6
	Minimum	25.2	25.5	26.9	30	21.9		26.8	27.4	24.6	31.7	23.7
PB 840	Maximum	24.9	25.1	26.1	30.2	30.5	PB 840	24.9	24.9	24.9	30	29.5
	Median	24.9	25.1	24.9	30.2	23.5	24.9	24.9	24	30	25.4	
	Minimum	24.9	25.1	20.5	30.2	20.8	24.9	24.9	21.8	30	23.3	
SAVINA	Maximum	25	27	26	30	30	SAVINA	26	26	26	30	30
	Median	25	27	25	30	25		26	26	25	30	25
	Minimum	25	27	23	30	23			26	22	30	22
SAVINA AF	Maximum	28	28	30	33	32	-	-	-	-	-	
	Median	28	28	28	33	27						
	Minimum	28	28	26	33	26						

Palv - Alveolar pressure at end-inspiration; VCV - volume-controlled ventilation; ICU - intensive care unit; RR - respiratory rate; Pmus - muscle pressure; PCV - pressure-controlled ventilation.

* Absence of muscle effort: Pmus = zero. Inspiratory effort: Pmus =-5cmH_2_O, Expiratory effort: Pmus = +5cmH_2_O. The spontaneous respiratory rate of the patient was set at 20bpm.

**Table 3:** Alveolar pressure at end-expiration or effective positive end-expiratory pressure in the volume-controlled ventilation and pressure-controlled ventilation modes for all ventilators and simulated scenarios

VCV mode ventilators ICU	Alveolar pressure at end-expiration or PEEPe						PCV mode	Alveolar pressure at end-expiration or PEEPe				
	Patient effort	No effort*	Pmus-5cmH_2_O		Pmus-5cmH_2_O/+5cmH_2_O			No effort*	Pmus - 5cmH_2_O		Pmus-5cmH_2_O/+5cmH_2_O	
	Ventilator RR	zero	15	25	15	25		zero	15	25	15	25
SERVO I	Maximum	10.1	10.2	10.4	10.3	10.2	SERVO I	10.1	10.3	10.6	10.2	10.4
	Median	10.1	10.2	10.4	10.3	8.3		10.1	10.3	10.4	10.2	8.4
	Minimum	10.1	10.2	7.4	10.3	7.2		10.1	10.3	7.7	10.2	7.2
ESPRIT	Maximum	10.4	10.7	12.3	10.6	15.8	ESPRIT	10.4	10.5	12.6	10.7	15.8
	Median	10.4	10.7	11	10.6	10.3		10.4	10.5	11.1	10.7	10.4
	Minimum	10.4	10.7	7.2	10.6	8		10.4	10.5	7.4	10.7	7.4
DX 3012	Maximum	10.4	10.6	12.1	10.7	14.8	DX 3012	10.4	10.7	12.1	10.7	12.8
	Median	10.4	10.6	10.8	10.7	10.1		10.4	10.7	11.2	10.7	10.2
	Minimum	10.4	10.6	7.6	10.7	7.6		10.4	10.7	8.2	10.7	8
PB 840	Maximum	10	9.9	10.1	9.9	9.9	PB 840	10	10	10.1	9.9	10
	Median	10	9.9	9.9	9.9	8.8		10	10	9.9	9.9	8.5
	Minimum	10	9.9	8.4	9.9	8.6		10	10	8.1	9.9	7.8
SAVINA	Maximum	10.4	10.8	12	10.7	12.7	SAVINA	10.7	10.6	12	10.7	12.7
	Median	10.4	10.8	11.2	10.7	11		10.7	10.6	11.1	10.7	11
	Minimum	10.4	10.8	10.9	10.7	9.7		10.7	10.6	10.9	10.7	9.5
SAVINA AF	Maximum	10.7	10.6	12.8	10.6	12.6		-	-	-	-	-
	Median	10.7	10.6	11.3	10.6	10.8						
	Minimum	10.7	10.6	11.2	10.6	9.8						

PEEPe - effective positive end-expiratory pressure; VCV - volume-controlled ventilation; ICU - intensive care unit; RR - respiratory rate; Pmus - muscle pressure; PCV - pressure-controlled ventilation.

* Absence of muscle effort: Pmus = zero. Inspiratory effort: Pmus =-5cmH_2_O, Expiratory effort: Pmus = +5cmH_2_O. The spontaneous respiratory rate of the patient was set at 20bpm.

**Table 4:** Transpulmonary pressure end of inspiration in the volume-controlled ventilation and pressure-controlled ventilation modes for all the ventilators and simulated scenarios

VCV mode ventilators ICU	Palv - Pmus						PCV mode	Palv - Pmus				
	Patient effort	No effort*	Pmus-5cmH_2_O		Pmus-5cmH_2_O/+5cmH_2_O			No effort*	Pmus - 5cmH_2_O		Pmus-5cmH_2_O/+5cmH_2_O	
	Ventilator RR	zero	15	25	15	zero		25	15	25		
SERVO I	Maximum	26.1	27	27.4	26.3	28	SERVO I	26.4	27.4	29	26.3	29
	Median	26.1	27	26.3	26.3	26.7		26.4	27.4	26.8	26.3	26.2
	Minimum	26.1	27	26.3	26.3	24.6		26.4	27.4	26.5	26.3	25.8
ESPRIT	Maximum	24.2	24.7	25.9	24.7	25.6	ESPRIT	24.1	25.7	27.5	27.1	28.2
	Median	24.2	24.7	24.9	24.7	24.5		24.1	25.7	24.3	27.1	26
	Minimum	24.2	24.7	24.6	24.7	23		24.1	25.7	24.3	27.1	25.4
DX 3012	Maximum	25.2	25.5	26.8	25	25.4	DX 3012	26.8	27.4	29.8	26.4	28.4
	Median	25.2	25.5	26.2	25	24.9		26.8	27.4	27	26.4	26.7
	Minimum	25.2	25.5	25.3	25	24.1		26.8	27.4	26.7	26.4	26.4
PB 840	Maximum	24.8	25	26	25	25.6	PB 840	24.8	24.8	26	25	27.8
	Median	24.8	25	24.8	25	24.6		24.8	24.8	24.3	25	25.7
	Minimum	24.8	25	24.6	25	22.4		24.8	24.8	23.8	25	25.4
SAVINA	Maximum	25.3	27	27.5	25.6	28	SAVINA	26.5	26.5	27.6	25.6	27.4
	Median	25.3	27	26	25.6	25.4		26.5	26.5	26.1	25.6	25.2
	Minimum	25.3	27	25.3	25.6	25.4		26.5	26.5	25.5	25.6	25
SAVINA AF	Maximum	28.6	28.4	30.8	28.5	30.7		-	-	-	-	-
	Median	28.6	28.4	29.7	28.5	27.8						
	Minimum	28.6	28.4	27.8	28.5	27.4						

Palv - Alveolar pressure at end-inspiration; Pmus - muscle pressure; VCV - volume-controlled ventilation; ICU - intensive care unit; RR - respiratory rate; PCV - pressure-controlled ventilation.

* Absence of muscle effort: Pmus = zero. Inspiratory effort: Pmus =-5cmH_2_O, Expiratory effort: Pmus = +5cmH_2_O. The spontaneous respiratory rate of the patient was set at 20bpm.

VT - tidal volume; VCV - volume-controlled ventilation; PCV - pressure-controlled ventilation; Pmus - muscle pressure; RR - respiratory rate.

### Assisted breaths with inspiratory efforts only

There were no trigger asynchronies when only inspiratory efforts were present (Pmus =-5cmH_2_O) and the mandatory RR was set to a lower value (15 bpm) than the simulated spontaneous RR of the patient (20 bpm). When triggered by the simulated patient, the median VT was 27mL lower than the set VT (range - 63 to +79mL) considering all five ventilators, and there was a variation in alveolar pressure at the end of inspiration, with a median of 25.4cmH_2_O (range 20.5 and 30cmH_2_O). The transpulmonary pressure at the end of inspiration increased with the variation in VT (24.3 to 29). The tidal volume variation, in this case, was due to asynchronous events.

### Assisted breaths with inspiratory and expiratory efforts

The results in the simulated scenarios with both spontaneous inspiratory and expiratory muscle efforts (Pmus =-5cmH_2_O followed by +5cmH_2_O) were as follows: first, with a mandatory RR lower than the simulated patient’s efforts, the median VT was higher than controlled breathing, but it was lower than the cases observed in the assisted MV when only inspiratory efforts were present in 6 of the 11 ventilator settings. On the other hand, Palv increased significantly above 28cmH_2_O, and the transpulmonary pressure end of inspiration values was similar to those obtained when only the inspiratory effort was present; second, VT increased significantly in 7 of the 11 settings (median value of 429, maximum 546 and minimum 367) with a mandatory RR higher than the spontaneous one, while the Palv, total PEEP and Palv-Pmus values showed the greatest variation among the simulated scenarios but with median values even lower or similar to those obtained in the situation of lower RR setting with synchronous assisted breathings.

These alterations were related to two factors. In the case with lower mandatory RR, the presence of expiratory muscle effort reduced or attenuated the increment in VT in relation to the inspiratory effort. In the second case, the presence of expiratory effort combined with higher mandatory RR resulted in patient-ventilator asynchronies.

In the scenario with the AF function active in the VCV mode of the Savina 300 ventilator, the VT was significantly higher than the VT measured when this function was inactive.

Similar VT, Palv, PEEP, and Palv-Pmus values were observed when VCV and PCV modes were compared in the same ventilator.

Interestingly, the effective PEEP remained at approximately 10cmH_2_O in most scenarios, except when the mandatory RR was set higher and when both inspiratory and expiratory efforts were present, which resulted in both increments and decrements (under pressurization). Only the Savina 300 ventilator with the AF function active in the VCV mode (VCV-AF) did not show decrements in PEEP below the value set.

## DISCUSSION

The main findings of the present study can be summarized as follows: assisted breaths resulted in a higher VT than those of controlled breathing cycles and in higher alveolar pressures, even in synchronic breathings; triggering asynchronies caused by setting the RR to a level higher than that of the patient’s spontaneous RR, regardless of the ventilatory mode - VCV or PCV - or the type of ICU ventilator, caused huge variations in VT and alveolar pressures at the end of inspiration; and early active expiratory effort during mechanical inspiration may limit VT augmentation and the correspondent increase in the alveolar distending pressures when there are no triggering asynchronies. On the other hand, when triggering asynchronies were present, the combination of inspiratory and expiratory efforts caused huge variations in VT, alveolar distending pressures, and PEEP, including over- and under-pressurization of the last parameter. The AF system of the VCV mode was associated with higher VTs and alveolar pressures.

Our results confirm previous findings demonstrating the effects of assisted breathing on the amount of VT and the pressure delivered to the lungs.

Morais et al.^(^18^)^ studied an experimental model of severe ARDS using mechanically ventilated rabbits and pigs and observed that muscle effort increased lung injury, especially in the dependent lung, where greater stress and local pulmonary stretch were generated. This phenomenon was minimized by using high PEEP, which may offset the need for muscle paralysis. Moraes et al.^(^30^)^ studied an experimental model of mild to moderate ARDS and found that high VT was associated with VILI and that VT control appeared to be more important than RR control to attenuate VILI. In the present study, in situations of inspiratory and expiratory muscle effort, VT was above 6mL/kg only in situations with the RR set at 25 breaths/min. However, when the RR was set at 15 breaths/min for the same effort pattern, there was an increase in alveolar pressure without an increase in VT. Our hypothesis for this result is that the presence of expiratory muscle effort had a limiting effect on VT, thereby preventing the value from exceeding 6mL/kg. Biehl et al.^(^31^)^ emphasize that patient-ventilator asynchrony often limits the use of low VT in ARDS patients requiring high minute ventilation, where adjustments of ventilator settings and sedative agents are modestly effective in limiting asynchrony, often requiring the use of neuromuscular blockade. The present study showed that in the mechanical model, simulated neuromuscular blockade, inspiratory muscle effort, and inspiratory/expiratory muscle effort had similar effects on VT variation when the model used an RR lower (RR at 15 breaths/min) than that of the ventilator (RR at 25 breaths/min). In addition, different patterns of VT variation and pulmonary pressures were found only in conditions where muscle effort was associated with RR higher (25 breaths/min) than that of the ventilator (RR at 20 breaths/min). Thus, it is reasonable to consider that the presence of muscle effort does not necessarily potentiate lung injuries due to excessive VT.

Respiratory rate setting is a key parameter in the management of MV, especially in patients who develop ARDS. Studies have reported that most patients with respiratory failure require a rate between 20 and 30 cycles/min, according to their needs.^(^8^,^31^-^33^)^ However, experimental studies with animals have shown that a higher RR may intensify VILI and that ventilated lungs with a lower RR produced less edema and perivascular hemorrhage than those ventilated with a higher RR.^(^34^)^ The results of the present study corroborate these findings, as setting the RR in the ventilator to a value above the patient’s RR generated variations in VT and pulmonary pressures, including values above the limits considered safe for the protective ventilatory strategy. It should be noted, however, that the study used a mechanical model in which the patient’s RR had a fixed pattern and did not vary according to their metabolic needs. Richard et al.^(^16^)^ compared a bench study with an in vivo study and showed that in both the mechanical and patient models, VT and its variability seemed to be influenced by the relationship between the patient’s RR and the RR setting in the ventilator -, i.e., the higher the rate, the lower the possibility of synchronous breathing cycles. In addition to highlighting the importance of adjusting VT, these findings also demonstrate the influence of RR on the variability of this ventilatory parameter because adjusting the RR of the ventilator to a value above the RR of the patient generates variations in VT and pressures since there is a respiratory effort by the patient.

Plateau pressure or alveolar pressure cannot and should not be considered a surrogate for pulmonary stress, as there is evidence of similar stress values for completely different VTs.^(^35^,^36^)^ In the present study, the values of alveolar pressure at the end of inspiration, until recently described in the literature as a predictor of lung injury when above 30cmH_2_O, only increased to this limit when there was Pmus-5/+5cmH_2_O with RR set at both 15 breaths/min and 25 breaths/min, especially in the PCV mode.

Briel et al.^(^37^)^ found that the reduction in VILI-related atelectrauma is associated with the optimization of PEEP values. However, the appropriate level of PEEP remains a matter of controversy. Randomized clinical trials, multicenter studies, and meta-analyses have not confirmed that PEEP above 12cmH_2_O reduces the mortality of ARDS patients.^(^38^-^40^)^ However, it is known that a very low end-expiratory lung volume may be related to cyclic opening and the collapse of unstable alveolar units. In this context, the detrimental effects of ventilation can be alleviated by the application of PEEP to prevent cyclic derecruitment of alveoli. However, PEEP should not be high enough to lead to excessive inflation. In the present study, PEEP levels remained close to the values of 10cmH_2_O in both modes, except for conditions where there was asynchrony, in which PEEP reached values higher than those set when Pmus-5cmH_2_O, thus suggesting hyperinflation due to the presence of auto PEEP, and values lower than those set when Pmus-5/+5cmH_2_O, thus suggesting system depressurization.

According to Lasocki et al.,^(^28^)^ the AF system is based on an attractive principle: it seeks to ensure adjusted VT while maintaining the advantages of PCV. Despite this potential advantage, clinical trials have not been conducted, and its clinical efficacy compared with conventional VCV has not been formally demonstrated.

In the present study, the use of the AutoFlow® system showed no advantages for patient-ventilator asynchrony compared with conventional VCV and PCV modes. In asynchronous situations, the VCV-AF mode delivered higher tidal volumes, thereby generating higher pulmonary pressures, which could potentially aggravate the development of VILI. The higher VT supply may be explained by the fact that the inspiratory flow generated in all experimental conditions in the VCV-AF mode was relatively higher than that in the other ventilatory modes.

In view of the complexity of studying critically ill patients with ARDS, the present bench study used an experimental model that aggregated variations in respiratory muscle effort patterns, ventilatory modes, and ventilator RR and allowed us to assess their impact on VT and pulmonary pressures during assisted MV in a mechanical ARDS model. Given the difficulties of conducting bedside studies, the ASL 5000™ lung simulator allows the development of studies using a very realistic simulation with good reproducibility and reliability and no risks for patients.

The clinical implications of this study include reaffirming the impact of the influence of muscle effort on VT variations and pulmonary pressures in ARDS patients; highlighting the importance of adjusting the ventilator’s RR, which is often neglected in clinical practice, thereby leading to patient-ventilator asynchrony; and emphasizing the importance of a careful choice of ventilatory mode and its management.

This study has some limitations. It used a mechanical model of the respiratory system. Bench conditions are not equivalent to patients whose efforts, pulmonary compliance, and respiratory system resistance can be highly variable, and the model had fixed inspiratory and expiratory Pmus and RR, i.e., the mechanical model did not react to a ventilatory demand, which prevented us from assessing patients’ physiological response to metabolic demands. Therefore, the results need to be confirmed in patients. Other limitations of the study are that patient-ventilator asynchronies were not evaluated, and compliance of the ventilator circuits was not measured, which may justify the VT difference between them.

## CONCLUSION

Adjusting respiratory muscle effort and pulmonary ventilator respiratory rate to a value above the patient’s respiratory rate in assisted/controlled modes generated large variations in tidal volume and pulmonary pressures, while the controlled mode showed no variations in these outcomes. On the other hand, the presence of expiratory muscle effort had a limiting effect on tidal volume and prevented the value from exceeding 6mL/kg. The pulmonary ventilator model influences ventilation even when similarly adjusted, which reinforces the need to standardize the pulmonary ventilator model in multicenter studies. The volume-controlled ventilation, volume-controlled ventilation with the AutoFlow® system, and pressure-controlled ventilation modes showed similar ventilation behavior. However, tidal volume and pulmonary pressures were slightly higher in the pressure-controlled ventilation and volume-controlled ventilation with the AutoFlow® system modes, thus suggesting that these modes require greater careful management during the use of protective mechanical ventilation with low tidal volume regulation.
